# Expansion and Contraction in Plaster and Vulcanite

**Published:** 1917-01

**Authors:** Stewart J. Spence

**Affiliations:** Chattanooga, Tenn.


### EXPANSION AND CONTRACTION IN PLASTER AND VULCANITE.

BY STEWART J. SPENCE, D.D.S., CHATTANOOGA, TENN.

My object in this paper is to give in condensed form the features of my writings scattered through the dental literature of the past thirty-three years on the subject of prosthesis, omitting those on celluloid and articulation, and touching but lightly on those on modelling compound, referring chiefly to those on plaster and vulcanite.

In the year 1882, while practicing in San Francisco, I began experimenting with plaster of Paris, with the object of finding how to get a minimum of expansion and maximum of hardness. At that time Richardson's "Mechanical Dentistry" was the standard, and it had taught that long-stirring made a cast hard and "tough." I tested this, and found that long-stirring largely increases expansion (and hastens setting) without increasing hardness or "toughness." I also found that dental plaster, being fine-grained, set with less expansion than plasterer's plaster, but softer, also that an addition of marble-dust made a cast softer without otherwise affecting it; also that alum was to be preferred to salt for hastening setting for casts, because doing so without inducing softness; and also that a slow-setting plaster, other things being equal, sets with least expansion. But I found no way of entirely eliminating plaster's two radical evils, softness and expansion; and the practical lessons, from that series of experiments were but these—Use a slow-setting dental plaster for impressions, and a slow-setting plasterer's plaster for casts and investments, both mixed to medium consistency, and with very short stirring.

In the year 1900 I took up again the study of plaster. My previous experiments had nearly all been made in celluloid blanks, but now I made them in impression trays, considering this the more scientific, because the tray is the thing in which the impression sets, and to this day I still believe it to be the most scientific instrument for testing the expansion and warpage of plaster. The celluloid blanks had invariably shown a space between blanks and cast at the palatal dome, but the impression tray sometimes failed to do this. On thinking this over, it occurred to me that perhaps this fact was due, not to the good behavior of the plaster in the tray by omitting to expand, but to the fact that the flange of the tray, being much more easily bent outward than that of the blank, had been slightly sprung outward by the plaster. This could be easily tested, for if the cast were removed from the tray so that the flanges would be permitted to spring back again, and the cast then refused to sit back in the tray, but rode upon the retracted flanges, showing a space at the palatal dome, this would prove it. On trial, this was found to be the fact. The unyielding flanges of the celluloid blank had, by resisting the lateral expansion of the plaster, forced it to find relief by bowing up at the palatal dome, there showing a space between blank and plaster. Not always, however, does this fail to occur in the tray; often there is a space between plaster and tray even before they have been separated, this showing that the resistance offered by the tray's flanges has only partly succeeded in preventing lateral expansion, the residue of the plaster's activity finding expression in a bowing up of the palatal surface, which may be termed warpage.

From these considerations I think it ought to be evident that there is no such thing as the impression expanding inwards, as Dr. Haskell and Dr. G. H. Wilson have taught. I have great respect for both these high authorities, but must differ with him here.

At the National Dental Association's Convention of 1903, I demonstrated this bowing up of plaster by showing a strip of plaster about thirteen inches long by three inches wide and half inch thick, which had been poured on a board, at each end of which was firmly screwed down a cleat to prevent lateral expansion. As a result, the strip was bowed up until at its highest point it was raised about a fourth-inch above the board. When the board was not oiled the adhesion of the plaster to it lessened this upward bulge, which led me to think that a thorough roughening of the surface of the tray would prevent to some extent bulging of a plaster impression. I also hit upon taking impressions in trays with removable flanges. As a matter of fact, however, I have long ago ceased to use plaster of Paris for impression-taking, and now use either a non-expanding plaster of my own invention or modelling compound. The contraction of the compound is very great if it is plunged into very cold water, but at the ordinary temperature of an office it is not enough to be a disadvantage, except perhaps in the case of a very hard mouth, in which case it should be kept warm until cast is poured. In many mouths this contraction is really an advantage.

I was much surprised at the time I was publishing my views on plaster's expansion and the value of curbing it, to find that some of our leading prosthetists either ignored or denied altogether the expansion of plaster of Paris. One large Supply House sent me a cast of the plaster they were retailing, to "prove that it had no expansion." I found that, on the contrary, when the cast had been shaken loose from the tray and then replaced therein, I could see a space between plaster and tray at a distance of about twelve feet and could insert into it thirteen folds of No. 20 tin foil. But a year or two later, Professor Prothero made some exhaustive experiments in plaster by which he arrived at conclusions very similar to mine, differing only in making the expansion somewhat less than I had. He agreed with me, however, that whether more or less, the expansion is enough to be a serious evil. Dr. Geo. H. Wilson has published in the second edition of his "Dental Prosthetics" a very full record of his experiments with various plasters, showing expansion, etc. It is, however, of little real value to attempt to decide the exact expansion of plaster, for no two barrels of the same brand give exactly similar results. It is enough to know that they all expand, which I have demonstrated by thousands of experiments, never having once failed to get expansion with undoctored plaster of Paris.

I also discovered that plaster of Paris has a second expansion. The first is over in about fifteen minutes, or as soon as the cast reaches its greatest warmth, but the second expansion progresses slowly for about thirty hours, and is only about a fourth as great as the former. By filling a plaster of Paris impression before this later expansion takes place, a small gain is made in the dimensions of the cast; but it is almost impossible to get ahead of the first expansion of the impression in the matter of pouring the cast, especially if the impression plaster is quick setting. Indeed, an impression plaster which sets fast enough to become more than just perceptibly warm to the fingers while setting in the mouth should be rejected, for its expansion will be great.

I also discovered later that plaster of Paris has a third expansion. This occurs when it is exposed to steam or water at the heat used in vulcanizing. Perhaps we have all noticed that in repairing an old vulcanite plate, nodules of the old vulcanite are found squeezed into air holes in the plaster; also that rubber is sometimes found squeezed out of the flask after vulcanization when none was seen there before. I had accepted the theory that such nodules and excess had been due to a tendency in rubber to swell under heat, but on observing that such nodules and excess rarely occur when a non-expanding plaster is used for cast and investment, I came to the conclusion that it was not the rubber, but the plaster which swelled. This guess was confirmed by experiments in which plaster casts, that had been poured in impression trays, were found to have taken on a large expansion in the vulcanizer. Other experiments showed that most of this expansion occurs by about the time the heat has reached 320 F., and this while the rubber is not yet hardened by vulcanization, but very soft from the high heat and the cast is but little softened, so that the rubber offers but slight resistance to the expansion of the plaster. To test this point I made a cast in a tray, then vulcanized rubber on it just as we do in making a plate, and found that when the cast was once more returned to the tray its expansion had very considerably increased. With dry heat this swelling did not occur; but it is very difficult to vulcanize with dry heat, as plaster parts with its moisture so slowly that it takes about an hour to reach 212 degrees F., after which the heat rises rapidly and is very hard to hold at 320 degrees. Besides, as dry heat shrinks plaster, the shrinkage is greatest where the heat is greatest, that is, near the outer parts of the cast, and thus warpage occurs.

Thus plaster of Paris casts have three expansions, besides a probable warpage. I know of no remedy for these evils; but as their tendency is to throw undue bearing of the plate on the palatal dome, a relief chamber partly counteracts this undue pressure; and as their tendency also is to widen the plate by lateral expansion of the impression and cast, so as to produce insufficient bearing on the buccal surfaces, the fit of the plate can sometimes be improved by scraping these buccal surfaces on the cast. But as there is now within the reach of all a plaster which has none of these eccentricities of plaster of Paris, it is but waste of time to try to counteract them by such clumsy methods as cast-scraping, etc. Besides, even were there some easy way of preventing the expansion of plaster of Paris, this would be only like locking the front door and leaving the back door open, for it would remedy but one of the two radical defects of plaster of Paris, namely, softness.

The softness, or compressibility, of plaster of Paris, is doubtless the cause of as many misfits as expansion. When, after being on the lookout for it nearly twenty years, I found a way to prevent the expansion of plaster of Paris, I fondly thought that from thenceforth I should have naught but good sailing in the matter of adaptation. But alas, I was sorely disappointed. I had to commence again at the foot of the ladder. Fortunately I remembered that in some experiments I made in celluloid many years before, I had found that the plaster casts were crushed out of shape badly in all cases where the celluloid was moulded at a low heat, and it occurred to me that possibly rubber, though so much softer than celluloid, might act similarly on casts, though to a lesser degree. I made an experiment to test this, and was surprised at the result. The compression of the plaster by the rubber was much greater than I had expected to find it; and on seeking for the cause it occurred to me that the rubber in a closing flash is *not* soft, but greatly increased in density and hardness by the compression. To test the amount of its compression of the cast I poured plaster into a swaged aluminum plate, then invested same in flask, and on opening flask and removing the aluminum plate, put rubber in its stead and closed flask in boiling water. Then on opening the flask and removing the rubber from the cast, I placed the cast back in the plate and found that it had largely been compressed out of its original shape. This became more and more obvious as I cut away the cast gradually, beginning at its posterior palatal border, and thus saw where the compression had ben greatest. I found that it was most compressed farthest from the borders, but that the greatest space was at a point not far from the border where an extra amount of rubber had been placed.

This experiment showed that plaster does not spread readily enough to avoid the evil of cast-compression. I set about trying to remedy this evil. I first thought it might be done by partially dissolving the rubber before closing the flask. I had noticed that a piece of rubber which dropped on some coal-oil on the work bench became softened thereby, and on trial in the flask I found that it permitted very easy closure of the flask; but, alas, on vulcanizing that rubber I found it was spoiled. I tried a number of solvents, from coal oil to chloroform, but they all injured the vulcanite. When chloroform was given an hour or so to evaporate, this injury did not occur, but by that time the rubber was no longer soft.

It occurred to me that perhaps the rubber was not given time enough in the hot water to become well softened; and I tested this by investing a thermometer in the plaster investment, and found, with surprise, that it required about fifteen minutes to reach 212 degrees F. This proved that plaster is a poor conductor of heat. Of course, the rubber could not take up heat before the plaster in contact with it did so. I made some tests to determine the extent of compression at different points of time of the flask-closing, and found that nearly all of it occurs at about the last turn of the nut. This taught me that if the nuts turn with unusual resistance near the end of flask-closing, the flask should be opened and some rubber removed.

This last lesson may be of value to those who use plaster of Paris for casts, but I no longer depend on it for that purpose, for I found a way to make non-expanding plaster so hard that especial care in flask-closing is not needed. I had been obsessed with the belief that a thick-mixed plaster set no harder than one mixed of medium consistency. My immature experiments in the San Francisco days had seemed to show this, but now I thought I would again put it to the test, and to my surprise and delight I found that the thicker I could mix my plaster the harder it set. Testing its resistance to compression when thus thickly mixed, against that of plaster of Paris, by filling one section of a flask with the one plaster and the other section with the other plaster, then placing a round steel ball between them and then closing the flask, I found that the ball sank from three to eight times more into the plaster of Paris than into my plaster. My method of measuring the respective displacements of plaster was somewhat crude, but sufficiently accurate for my purpose. It consisted in filling the two holes with wax, then taking out the wax and rolling it into two equally thick ropes, then measuring these. Dr. Geo. H. Wilson has since made some very exact measurements of the resistance of various plasters, mine included, as well as their expansions, times of setting, etc., recording the same in his second edition of "Dental Prosthetics."

With a non-expanding and virtually non-compressible cast, I thought I had at last solved the problem of adaptation; but the end was not yet. I was still using plaster of Paris for investments, and the fits were not always ideal. Where could the fault be? Pondering over the subject, I remembered that I had read where Dr. G. B. Snow said that vulcanite contracts. By testing this, I found that a strip of vulcanite would contract, when perfectly free to do so, as much as one-sixteenth of an inch in three inches when heated to 320 degrees F. This much at the first contraction, and more at a second and third similar heating, until about a twelfth-inch contraction was reached. Of course such great contraction would ruin any plate; but as flasked rubber is not free to contract, being resisted by the investment, no such vast contraction occurs in actual practice. The question arose, "Does a plaster of Paris investment prevent all contraction?" A few experiments showed that it did not, and also that investments of plaster of Paris which were given only an hour or two to harden did not resist contraction nearly so well as those given all night. Experiments further showed that it is during the cooling that this contraction occurs, at which time the plaster investment has reached its softest state. If this contraction were equal all over the plate it would result merely in an all around smaller plate (which might be an advantage) but this can not occur, because some parts of a plate are thicker and therefore stronger to contract than other parts, and also because the investing plaster is apt to be harder at some parts than others and therefore to grip the plate tighter at these parts. It seemed likely that this last happens at one side or other of the plate, for my tests showed that one wing of a plate will often be found drawn down, so as not to sit on the test cast at that side of the ridge while sitting all right on the other side. In my early days I had a case where the plate split asunder inside the vulcanizer with a loud snap, the crack running for about an inch along the median line from back to front, with one of the wings warped inwards about a quarter of an inch. The plaster investment of this case had been made of dental plaster, which sets softer than coarser plaster, and, as it was a hurry job, it had not been given time to set hard. It was almost mushy in the vicinity of the crack. This illustrates that warpage of plate may result from contraction of vulcanite.

When this warpage occurs, the plate is spoiled as regards fit, but it may sometimes be remedied by immersion in hot water. This has a tendency to restore a vulcanite or celluloid plate to the shape in which it was moulded. But, as heat also causes contraction, there is great danger of shrinking the plate too much, to prevent which the heating of it should be raised by gradual steps, and at each step the plate tried in the mouth. My experiments demonstrated that vulcanite contraction begins at 130 degrees F., but some very loose and over-large plates may be raised to 180 degrees F. or even 190 degrees F. before becoming too small. Thus misfitting plates can sometimes be made into good fits. A theremometer should be placed in the water with the plate.

It is well to apply this shrinking method to plates which come in to be refitted, especially thick plates, thus reducing their vertical thickness in advance.

This leads me to my last discovered source of misfit, and its remedy. I failed to get a good adaptation in a plate which had great thickness of vulcanite over the ridges. This set me to wondering. It then occurred to me that the shrinkage of vulcanite might be the evil-doer. This did not appeal to me at first, but when I reflected that vertical shrinkage (that is, contraction of the plate's thickness) is not restrained as much by the investment as it is a real shrinkage (that is, contraction of the plate's area), if indeed it is restrained at all, I became convinced that we have here a fruitful source of mal-adaptation. For as three inches of vulcanite will contract a sixteenth or twelfth of an inch, it follows that a half-inch will contract about a ninetieth of an inch, which is quite an item when deducted from the thickness over the ridge and therefore added to that over the palatal dome. My first attempts at preventing this vertical shrinkage were crude indeed, consisting in drilling holes in the case, which, when filled with vulcanite, would hold the walls of vulcanite to the cast. The next method was the use of continuous gum teeth, the long and serrated roots of which would hold the vulcanite walls apart; which method gave way to serrated pieces of platinoid stuck into the rubber while packing, to answer the same purpose as the tooth roots. All these crude methods were supplanted by the final one, which consisted in using a non-contracting rubber to fill in the thick parts of plate. Having observed that weighted rubber shrank less than ordinary rubber, but still not enough to prevent all shrinkage, it occured to me to mix more chips of metal with said weighted rubber until absolute non-contraction should be reached, in making which experiments I noticed that the smaller my chips and filings, the better the result, so that it occurred to me that perhaps some metal reduced to a powder might work best. The lighest metal being aluminum, I chose it, and mixed the powder known as aluminum bronze with rubber by dissolving the rubber in chloroform and kneading the powder into the rubber. A few tests showed that from twenty to twenty-five per cent. of the metal by weight, gave absolute non-expansion to rubber. On cutting open a large lump of vulcanite thus treated, I was delighted to find that the aluminum had also prevented porosity. A small quantity of this bronze mixed with black rubber gives the pretty "gold dust" appearance, but twenty per cent. mixed with red rubber gives a steel grey shade not so pretty; therefore it is desirable to wrap the aluminized rubber in a fold of the red rubber. While the addition of the metal of course weakens the vulcanite, as does any other ingredient, yet as this rubber is intended only for thick parts of the plate, this slight decrease of strength is of no consequence. Any dentist can easily prepare this non-contracting rubber for himself. The chloroform must be given an hour or two to evaporate before using, else the vulcanite will be porous.—*Dental Digest.*

